# The TUITEK^®^ patient support program improved caregiver-related behaviors on growth hormone treatment adherence

**DOI:** 10.3389/fendo.2025.1548558

**Published:** 2025-04-28

**Authors:** Ekaterina Koledova, Pen-Hua Su, Yen-Ju Chen, Aria Assefi, Matias Debicki, Debbie Cooke, Amrit Jheeta, Alexander B. Jones, Jung Eun Moon

**Affiliations:** ^1^ Global Medical Affairs Cardiometabolic & Endocrinology, Merck Healthcare KGaA, Darmstadt, Germany; ^2^ School of Medicine, Chung Shan Medical University, Taichung, Taiwan; ^3^ Department of Pediatrics and Genetics, Chung Shan Medical University Hospital, Taichung, Taiwan; ^4^ Medical and Clinical Affairs Department, Merck Ltd., Taipei, Taiwan; ^5^ Medical Department, Merck S.A., Buenos Aires, Argentina; ^6^ Patient Support Program, Merck S.A., Buenos Aires, Argentina; ^7^ Atlantis Health Ltd, London, United Kingdom; ^8^ inScience Communications, London, United Kingdom; ^9^ Department of Pediatrics, School of Medicine, Kyungpook National University, Kyungpook National University Hospital, Daegu, Republic of Korea

**Keywords:** adherence behaviors, patient support program, recombinant human growth hormone, growth hormone deficiency, behavior change techniques

## Abstract

**Background:**

Recombinant human growth hormone (r-hGH) can improve or normalize growth outcomes in pediatric patients with growth hormone deficiency, but poor adherence to the treatment regimen limits treatment effectiveness. TUITEK^®^ is a multicomponent patient support program (PSP) designed to deliver support aimed at behavior change that is personalized to the needs of individual caregivers and patients throughout the treatment care pathway. The aim was to assess the impact of the TUITEK^®^ PSP on knowledge, beliefs and perceptions of adherence to r-hGH treatment in high-risk caregivers.

**Patients and methods:**

A prospective pre–post research was conducted across the combined population of caregivers of patients with short stature receiving r-hGH treatment in the TUITEK^®^ PSP in Argentina, South Korea, and Taiwan. Caregivers who were categorized as high-risk based on suboptimal knowledge, beliefs and perceptions of factors influencing adherence to r-hGH treatment (disease and treatment coherence, emotional burden, self-administration, and treatment-related anxiety) were included in the analysis.

**Results:**

In total, data from 409 caregivers were available. Involvement in the TUITEK^®^ PSP resulted in a statistically significant (p<0.0001) positive change for all factors. Improvements were reflected in the number of caregivers who moved from high- to low-risk at the end of the TUITEK^®^ PSP. The overall changes were reflected in the changes observed when data were analyzed for individual countries separately.

**Conclusions:**

The TUITEK^®^ PSP successfully improved key caregiver-related behaviors that may negatively impact adherence to r-hGH treatment and might improve adherence and therefore clinical outcomes.

## 1 Introduction

Recombinant human growth hormone (r-hGH) can improve or normalize growth outcomes in pediatric patients with growth hormone deficiency (GHD) and related conditions. However, poor adherence to the treatment regimen limits the overall effectiveness of r-hGH treatment (1) and has negative effects on long-term clinical, psychological and growth outcomes, as well as healthcare system costs and resource use (2, 3). In Argentina and Asia, 34% and 27% of patients, respectively, have been reported as having low-to-intermediate adherence rates (4, 5). These low adherence rates for r-hGH treatment may be the result of insufficient knowledge about the disease and its treatment, the need for daily injections from childhood to late adolescence, discomfort and pain associated with daily injections, treatment beliefs, treatment-related anxiety, a lack of self-efficacy, or concerns about long-term treatment (3). Furthermore, motivation may be low as the benefits are not immediately apparent (6), and there may be cultural and individual beliefs and attitudes that affect levels of adherence (7, 8).

In pediatrics, adherence to treatment is affected by the behaviors of both the patient and the caregiver (9), and educational and motivational interventions should, therefore, be targeted at both groups. The benefit of patient support programs (PSP) aimed at helping both patients and their caregivers recognize and manage behaviors that negatively affect treatment adherence have been successful demonstrated, improving adherence to r-hGH treatment (3). Those designed for caregivers, aimed at factors relating to their involvement and participation, including their sense of coherence, have been shown to improve adherence behaviors (10, 11).

Patient journey analysis has shown that early awareness training and treatment onboarding are key factors to establish a treatment routine and help patients deal with injection pain (12). This is best achieved by early access to patient support groups and the chance to build connections with other families with similar experiences which can foster understanding of the future patient journey. As part of this, healthcare professionals (HCPs) have a role in supporting patients and their caregivers to maintain ongoing care and wellbeing.

TUITEK^®^ is a multicomponent PSP designed to deliver support aimed at behavior change that is personalized to the needs of individual caregivers and patients, throughout the treatment care pathway. The TUITEK^®^ PSP is facilitated by trained nurse practitioners by telephone (including online systems) and uses behavior change techniques (BCTs) and motivational interviewing principles to address the issues that caregivers of patients with GHD encounter during r-hGH treatment (13). TUITEK^®^ was developed using a structured approach based on the behavior change wheel, with the Capability, Opportunity, Motivation, and Behavior (COM-B) framework used to identify the drivers of behavior and explore how a PSP could address them (13).

The program focuses on disease and treatment coherence, emotional burden, treatment-related anxiety and self-administration. Within the Taiwan TUITEK^®^ program, the emotional burden of the child, treatment necessity, and social support were also included. A personalization screening questionnaire, developed based on the outcomes of a literature review and behavior diagnosis (3), enables the program to be tailored to the needs of the caregiver and calls with a specialist nurse add a human element and may help to support engagement. The effectiveness of the TUITEK^®^ PSP has previously been demonstrated in small studies in Argentina, South Korea and Taiwan (2, 10, 14).

We aimed to assess the impact of the TUITEK^®^ PSP on knowledge, beliefs and perceptions of adherence to r-hGH treatment in high-risk caregivers in Argentina, South Korea, and Taiwan. High-risk was based on suboptimal knowledge, beliefs and perception factors influencing adherence to r-hGH treatment (disease and treatment coherence, emotional burden, self-administration, and treatment-related anxiety). Combining the data from the three countries provides a larger population to observe the effects in, as well as providing data across multiple different healthcare systems.

## 2 Materials and methods

A prospective pre–post research was conducted.

### 2.1 Participant recruitment and inclusion criteria

The caregivers (aged 25–60 years) of patients who were diagnosed with short stature due to GHD and who were undergoing treatment with r-hGH (somatropin; Saizen^®^, Merck KGaA, Darmstadt, Germany) using the Easypod^®^ auto-injector device or Aluetta^®^ injection pen (Merck KGaA, Darmstadt, Germany) in Argentina, South Korea and Taiwan were included in the program. Caregivers suffering from any mental illness were excluded from the program. High-risk caregivers were defined using a personalization questionnaire based on the four key factors: disease treatment and coherence, emotional burden, treatment-related anxiety and self-administration (5-point rating scales, see **Supplementary Tables S1**-**S3**) (3). The wording of the personalization questions differed by country due to translation and localization based on local experience and feedback provided by HCPs participating in the program. Scores of 1–3 for disease and treatment coherence and self-administration and 3–5 for emotional burden and treatment-related anxiety in the baseline personalization questionnaire were considered high-risk. This prospective research only evaluated participants who were classified as high risk based on being identified as high-risk for at least one of the key factors.

### 2.2 TUITEK^®^ PSP

The TUITEK^®^ PSP is delivered by nurse practitioners who have received a training session with the aim of developing key skills and strategies to effectively deliver the program, and a PSP manual containing a personalization questionnaire directed to caregivers to identify the priority topics, a scheduled contact guide, and resource packs incorporating BCT and motivational interviewing guides. Responses to the personalization questions determined the support needs for each participant and PSP nurses used a personalization algorithm to assess the priority topics for interventional phone calls. If the participant was scored as high-risk for disease and treatment coherence, this factor was prioritized. High-risk caregivers received up to five personalized calls over up to 12 weeks from a nurse practitioner. The calls aimed to address unhelpful beliefs and perceptions that caregivers may have about r-hGH treatment and to provide guidance on how best to support positive behavior change in both caregiver and patient. As Taiwan was the first location to conduct the TUITEK^®^ PSP, caregivers in this country could continue in the program for one year, and caregivers could be re-enrolled if they remained as high-risk for at least one of the key factors used for screening.

### 2.3 Data

Data were extracted up to 13 November 2023 for Argentina, 6 October 2023 for South Korea and 23 March 2023 for Taiwan. Caregivers were contacted two weeks after the final call of the TUITEK^®^ PSP to complete a follow-up personalization questionnaire to assess the changes in the questionnaire-based scores.

### 2.4 Statistical analysis

Data were analyzed for participants who scored as high risk for each question in the analyses related to that question. The baseline and follow-up questionnaire data were compared to assess the questionnaire-based scoring pattern of the perceptions and beliefs of the caregivers. In South Korea the program had separate items for disease coherence and treatment coherence, rather than a combined disease and treatment coherence question, so the average of each person’s disease coherence and treatment coherence questions were used to create a proxy disease and treatment coherence score.

Normality of the data was examined to check assumptions using parametric tests with GraphPad Prism. Due to the non-normal distribution of the data, a nonparametric Wilcoxon signed-rank test was performed to compare changes in the questionnaire-based scores between the paired groups at baseline and follow-up, overall and in each country separately. A p-value <0.05 (two-sided) was considered statistically significant.

### 2.5 Ethics

Ethical review and approval was not required for this research study in accordance with the local legislation and institutional requirements. All caregivers and patients consented to participate in the TUITEK^®^ program and written informed consent was obtained from eligible participants at the start of the program. All participants consenting to participate in the program agreed to share their data for analysis. The study used existing data that was presented in a deidentified manner with no identification of individual patients; data privacy and security were maintained throughout the study.

## 3 Results

In total, 409 high-risk caregivers (Argentina, n=222; South Korea, n=88; Taiwan, n=99) were available (**Table 1**). Out of these, 10.3% were considered high-risk for disease and treatment coherence, 71.4% for emotional burden, 35.9% for self-administration and 58.7% for treatment-related anxiety. When individual countries are considered, the proportion of caregivers at high-risk for emotional burden was lower for South Korea compared with Argentina and Taiwan (56.8%, 73.0% and 80.8%, respectively), and for self-administration was lower for Argentina compared with South Korea and Taiwan (14.0%, 62.5% and 61.6%, respectively). For the two other factors evaluated, disease and treatment coherence and treatment-related anxiety there were no major differences between countries for the proportion at high risk.

**Table 1:** Number and percent of caregivers scoring in high-risk category at baseline, with scores at baseline and follow-up for each factor.

		Initial number (%) of high-risk caregivers	Initial median score (IQR) [Range]	Final median score (IQR) [Range]
**Disease and treatment coherence^†^**	**Argentina**	17 (7.7)	3 (3–3) [1–3]	5 (4.5–5)* [4–5]
	**South Korea**	5 (5.7)	3.5 (3–3.75) [3–4]	4.5 (3.75–5) [3.5–5]
	**Taiwan**	20 (20.2)	3 (3–3) [1–3]	5 (4–5)* [3–5]
	**Overall**	42 (10.3)	3 (3–3) [1–4]	5 (4–5)* [3–5]
**Emotional burden^†^**	**Argentina**	162 (73.0)	3 (3–4) [3–5]	3 (3–3)* [1–5]
	**South Korea**	50 (56.8)	4 (3–4) [3–5]	2 (1–3)* [1–5]
	**Taiwan**	80 (80.8)	4 (3–5) [3–5]	2 (2–2.75)* [1–5]
	**Overall**	292 (71.4)	4 (3–4) [3–5]	3 (2–3)* [1–5]
**Self-administration^†^**	**Argentina**	31 (14.0)	3 (1–3) [1–3]	4 (3–5)* [2–5]
	**South Korea**	55 (62.5)	3 (1–3) [1–3]	4 (3–5)* [1–5]
	**Taiwan**	61 (61.6)	2 (1–2) [1–3]	4 (2.5–4)* [1–5]
	**Overall**	147 (35.9)	2 (1–3) [1–3]	4 (3–5)* [1–5]
**Treatment-related anxiety^†^**	**Argentina**	125 (56.3)	3 (3–4) [3–5]	3 (2–3)* [1–5]
	**South Korea**	52 (59.1)	3 (3–4) [3–5]	2 (1–2)* [1–5]
	**Taiwan**	63 (63.6)	3 (3–4) [3–5]	2 (2–3)* [1–5]
	**Overall**	240 (58.7)	3 (3–4) [3–5]	2 (2–3)* [1–5]
**Social support**	**Taiwan**	40 (40.4)	3 (3–4) [3–5]	2 (2–3) [1–4]
**Treatment necessity**		20 (20.2)	3 (2–3) [1–3]	4.5 (4–5) [3–5]
**Emotional burden (child)**		50 (50.5)	3 (3–4) [3–5]	2 (2–2) [1–4]
**Treatment-related burden**		22 (22.2)	3 (3–4) [3–5]	2 (1–2) [1–4]

Argentina, N=222; South Korea, N=88; Taiwan, N=99. *Statistically significant difference from initial value (p<0.0001). **^†^** Higher scores on disease and treatment coherence and self-administration, and lower scores on emotional burden and treatment-related anxiety implies better outcomes.

The mean (standard deviation) duration caregivers were in the TUITEK^®^ PSP (from the initial survey to define risk, to the final survey) in Argentina and South Korea was 43.4 (29.1) days and 43.7 (23.2) days, respectively. The median (range) durations were 34 (12–185) days and 43 (10–195) days, respectively. In Taiwan, where the TUITEK^®^ program has been ongoing for a longer period of time, the mean (standard deviation) duration caregivers were in the PSP was 366 (12) days and the median (range) duration was 369 (334–389) days.

**Figure 1:**
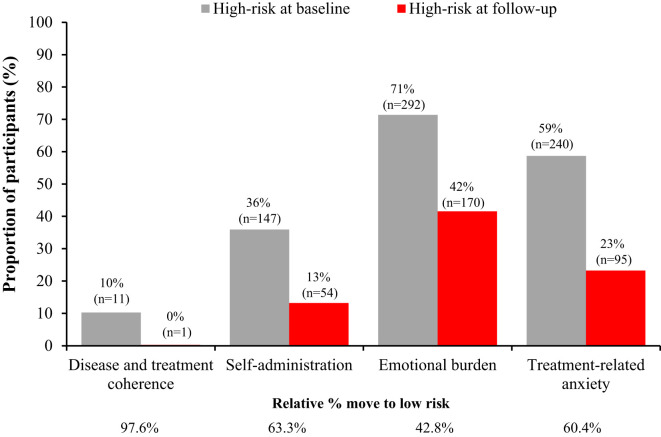
Distribution of patients at high-risk at baseline and follow-up for each factor in the TUITEK^®^ PSP. Data are only shown for patients at high risk at baseline, the remaining proportion of participants are those who are low risk at baseline.

When the overall data were evaluated, the median (interquartile range) score for each factor at baseline and after participation in the PSP are shown in **Table 1**. Out of 409 caregivers, 42, 147, 292 and 240 were categorized as high-risk for disease and treatment coherence, self-administration, emotional burden, and treatment-related anxiety, respectively. Involvement in the TUITEK^®^ PSP resulted in a positive change for all factors (p<0.0001). Improvements were reflected in the number of caregivers who moved from high- to low-risk at the end of the TUITEK^®^ PSP (**Figure 1**). The greatest change was seen for disease and treatment coherence, for which only one of the 42 high-risk caregivers did not move to the low-risk category.

**Figure 2:**
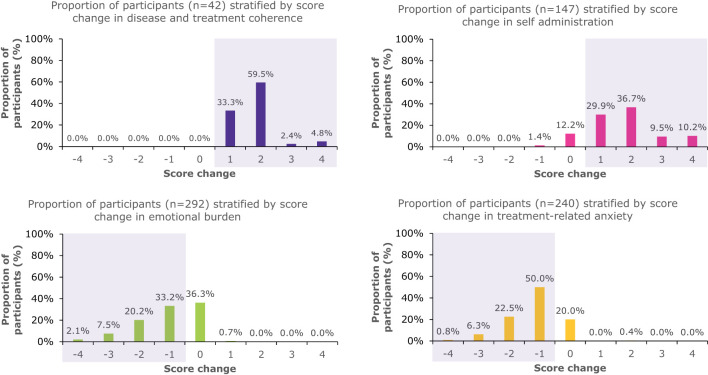
Proportion of patients who were high-risk at baseline who had score changes in the overall PSP population. The shaded areas indicate beneficial changes in score.

When looking at any positive change in score, 100% of caregivers who were high-risk at baseline had a beneficial change in disease and treatment coherence, 86.4% a change in self-administration, 63% a change in emotional burden, and 79.6% a change in treatment-related anxiety (**Figure 2**).

**Figure 3:**
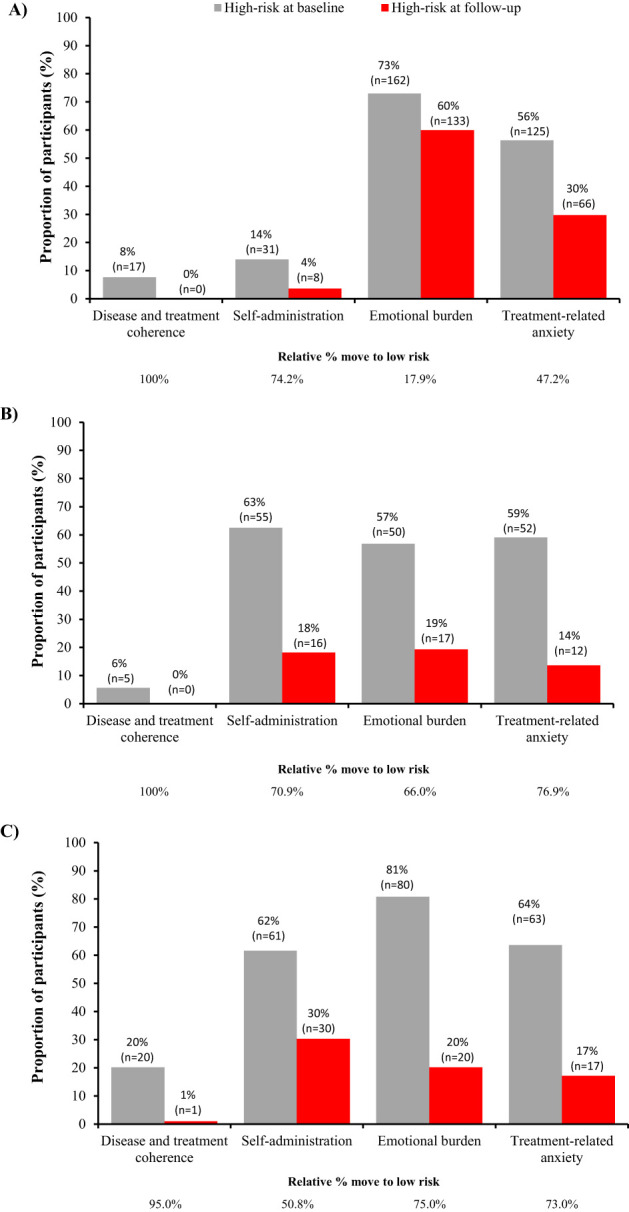
Distribution of patients at high-risk at baseline and follow-up for each factor in the TUITEK^®^ PSP in **(A)** Argentina, **(B)** South Korea, and **(C)** Taiwan. Data are only shown for patients at high risk at baseline, the remaining proportion of participants are those who are low risk at baseline.

Differences between individual country data were observed when these were evaluated separately (**Table 1**, **Figure 3**). All three countries exhibited similar performance for disease and treatment coherence, with 95–100% moving from high to low risk. However, for self-administration a greater proportion moved from high to low risk in Argentina and South Korea, compared with Taiwan (74.2%, 70.9%, and 50.8%, respectively). Further, a lower proportion of caregivers in Argentina moved from high to low risk for emotional burden and treatment-related anxiety (17.9% and 47.2%, respectively) compared with South Korea (66.0% and 76.9%, respectively) and Taiwan (75.0% and 73.0%, respectively). When the proportion of caregivers with any positive change in score was evaluated by country, a similar response pattern was observed for disease and treatment coherence, emotional burden and treatment-related anxiety (**Supplementary Figures S1**-**S3**). However, similar proportions of caregivers in South Korea and Taiwan showed no positive change in self-administration (15.7% and 16.4%, respectively) despite a greater shift to low risk in South Korea, reflecting the higher baseline scores in South Korea. The changes in median score and proportions of caregivers moving from high- to low-risk was consistent between South Korea, where data are reported for approximately 3 months in the program, and Taiwan, where data are reported for approximately one year in the program.

Four additional categories were included in the Taiwan TUITEK^®^ program, emotional burden (child), treatment necessity, social support and treatment-related burden (**Table 1**, **Supplementary Figures S4**, **S5**). Positive changes were seen for all of these categories with 84.0%, 75.0%, 62.5% and 71.8% of caregivers who were high-risk at the start of the program moving to low risk. This is reflected in the proportion of caregivers with any positive change in score.

## 4 Discussion

The TUITEK^®^ PSP was shown to be effective in addressing perceptions and emotional factors known to affect treatment adherence behavior among caregivers deemed at high-risk for factors relating to non-adherence. Addressing these factors, should, in turn, improve clinical outcomes although these were not assessed in this analysis of the PSP (3).

Disease and treatment coherence in this sample of caregivers was high at baseline, and remained high for the duration of the support program. At baseline, many caregivers did not perceive their child to be responsible for managing their own disease and/or did not perceive that their child was responsible for the administration of r-hGH. This perception improved greatly during the TUITEK^®^ PSP, and many caregivers considered that their child was responsible for managing their own disease by follow-up, suggesting a shift in responsibility of care, readiness for transition and a move towards empowerment. A strong emotional burden of caring for a patient on r-hGH treatment was evident in our study population, as demonstrated by high baseline scores. Emotional burden was reduced during the pilot, suggesting that the TUITEK^®^ BCTs supported caregivers to better manage the emotional impact they were experiencing. Many caregivers in our study had a high level of treatment-related anxiety at baseline, suggesting that this is a significant challenge for many people caring for a child with GHD. Over the duration evaluated, treatment-related anxiety levels were reduced. In Taiwan, the outcomes of the four additional categories evaluated were also positive, with coherence observed between the main outcomes evaluated on caregiver behaviors and the outcomes relating to the caregiver’s perception of their child’s behavior.

These results confirm the benefits of the multi-factorial approach utilized in the TUITEK^®^ PSP. They show, for example, that caregiver’s anxiety about the risk of side-effects of r-hGH treatment and the need for daily injections can be reduced by reassurance provided by the TUITEK^®^ PSP nurse practitioners. Additionally, because r-hGH treatment tends to begin at a very young age, caregivers can find it difficult to allow their child to take full responsibility for managing their own condition and self-administering their daily injections as their child gets older. The results of our research confirm that the TUITEK^®^ PSP can facilitate this process by reducing the anxiety the caregivers had about this transition process, enabling greater independence and autonomy of the child (15).

At baseline, a low proportion of caregivers were considered high-risk for disease and treatment coherence in Argentina and South Korea (7.7% and 5.7%, respectively). This is likely because there is high disease awareness in Argentina and South Korea (16). There was also a low proportion of caregivers in the high-risk category for self-administration in Argentina (14.0%). Differences between individual country data for some of the outcomes were observed when evaluated separately, with a greater proportion moving from high to low risk for self-administration in Argentina and South Korea, compared with Taiwan, and a lower proportion moving from high to low risk for emotional burden and treatment-related anxiety in Argentina compared with South Korea and Taiwan. This low proportion of patients moving from high to low risk in Argentina has also been observed in previous analysis of the TuiTek PSP in Argentina (2). These may relate to cultural differences or differences in the healthcare systems between the countries.

A different nurse-led educational intervention in Argentina for patients with low adherence, which was supported by digital medication adherence monitoring, found that median adherence 6 months after the targeted educational visit from a PSP nurse was significantly improved, with 36% of patients improving to ≥80% adherence (11). Studies in other chronic conditions have demonstrated the benefit of educational and motivational interventions to improve adherence behaviors (17, 18). These can also help alleviate some of the drivers of negative treatment experiences of caregivers which include the need to become experts in a condition, the burden of the mental load of managing treatment, overcoming misconceptions of care, and the need to battle for support (19).

Our results confirm the findings of the previous studies on the impact of the TUITEK^®^ PSP in Argentina, South Korea and Taiwan, showing that it can be effective across a variety of cultural and linguistic environments (2, 10, 14). Over one year in Taiwan, a greater proportion of caregivers moved from high to low risk for emotional burden, confidence of self-administration and treatment compared with an analysis over 3 months, demonstrating a continued longer-duration benefit of the intervention and supporting the continued benefit of the TUITEK^®^ PSP (10). Despite cultural and linguistic differences and differences in healthcare systems between the three countries involved in the PSP and differences in terms of disease awareness, treatment/clinical practice (20) and reimbursement (21, 22), we found improvements in all of the adherence parameters identified and addressed by the TUITEK^®^ PSP, confirming the potential value of this approach for all caregivers of children with GHD across diverse healthcare settings.

A strength of this analysis of the PSP was the inclusion of a larger number of caregivers than previous studies, which increases the robustness of the results. However, there were a number of limitations, including: the inclusion of data from only three countries, with the duration of exposure varying between countries; the short duration of follow-up included in the PSP, as the behaviors addressed may worsen when caregivers are not receiving bi-weekly contact, and longer-term nurse contact may be needed to ensure that the behaviors are improved over the longer-term; caregivers’ perspectives of risk can fluctuate over time and therefore provide only a snapshot of the days the questionnaires were administered; and we were not able to directly correlate the improvements observed in caregiver behaviors, attitudes and perceptions with adherence data. Therefore, future studies are required to determine whether the improvements observed in these outcomes with the TUITEK^®^ PSP are associated with improved adherence and other patient-reported outcomes such as quality of life.

In future studies, in which the Easypod^®^ device is used, its digitally enhanced features will enable adherence to be tracked, and this can be associated with data from the TUITEK^®^ PSP which can demonstrate the impact of the improvements observed in behaviors on real-world adherence. In addition, aspects of the TUITEK^®^ PSP are being digitalized in Latin America to provide support through additional channels and on-demand when caregivers feel they most need the support; this includes the use of short videos, personalized messages, chatbots and notifications. This digitization aims to enable longer-term support without excessively increasing the cost to deliver the program.

## 5 Conclusions

Our research demonstrates that the TUITEK^®^ PSP successfully improved key caregiver-related behaviors that may negatively impact adherence to r-hGH treatment. Our results support the findings of previous, smaller studies in Argentina, South Korea and Taiwan and suggest that the principles of the TUITEK^®^ PSP can be applied across a range of cultures and languages to support caregivers of children with GHD and assist the work of HCPs involved in the treatment of GHD.

## Data Availability

Any requests for data by qualified scientific and medical researchers for legitimate research purposes will be subject to Merck’s Data Sharing Policy. All requests should be submitted in writing to Merck’s data sharing portal https://www.merckgroup.com/en/research/our-approach-to-research-and-development/healthcare/clinical-trials/commitment-responsible-data-sharing.html. When Merck has a co-research, co-development, or co-marketing or co-promotion agreement, or when the product has been out-licensed, the responsibility for disclosure might be dependent on the agreement between parties. Under these circumstances, Merck will endeavor to gain agreement to share data in response to requests.
