# Supplementary material for: The TUITEK® patient support program improved caregiver-related behaviors on growth hormone treatment adherence
Source: Front Endocrinol (Lausanne). 2025 Apr 28;16:1548558. doi: 10.3389/fendo.2025.1548558 (PMC12066293; doi:10.3389/fendo.2025.1548558)
Supplement: Supplementary file 1 [file DataSheet1.docx]

# Supplementary Material

Supplementary Figure S1. Proportion of patients who were high-risk at baseline who had score changes in Argentina

**
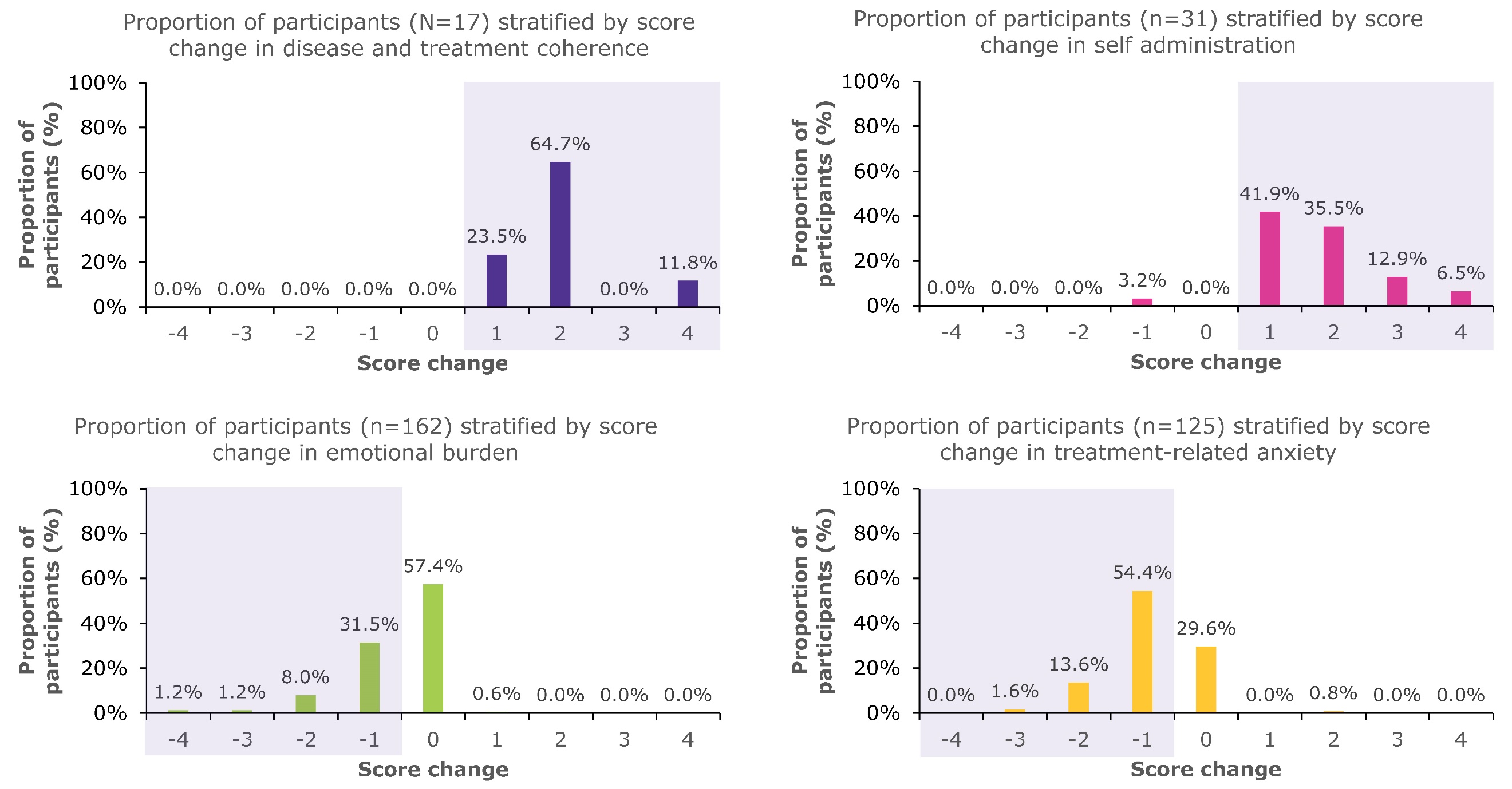
**

The shaded areas indicate beneficial changes in score

Supplementary Figure S2. Proportion of patients who were high-risk at baseline who had score changes in South Korea

**
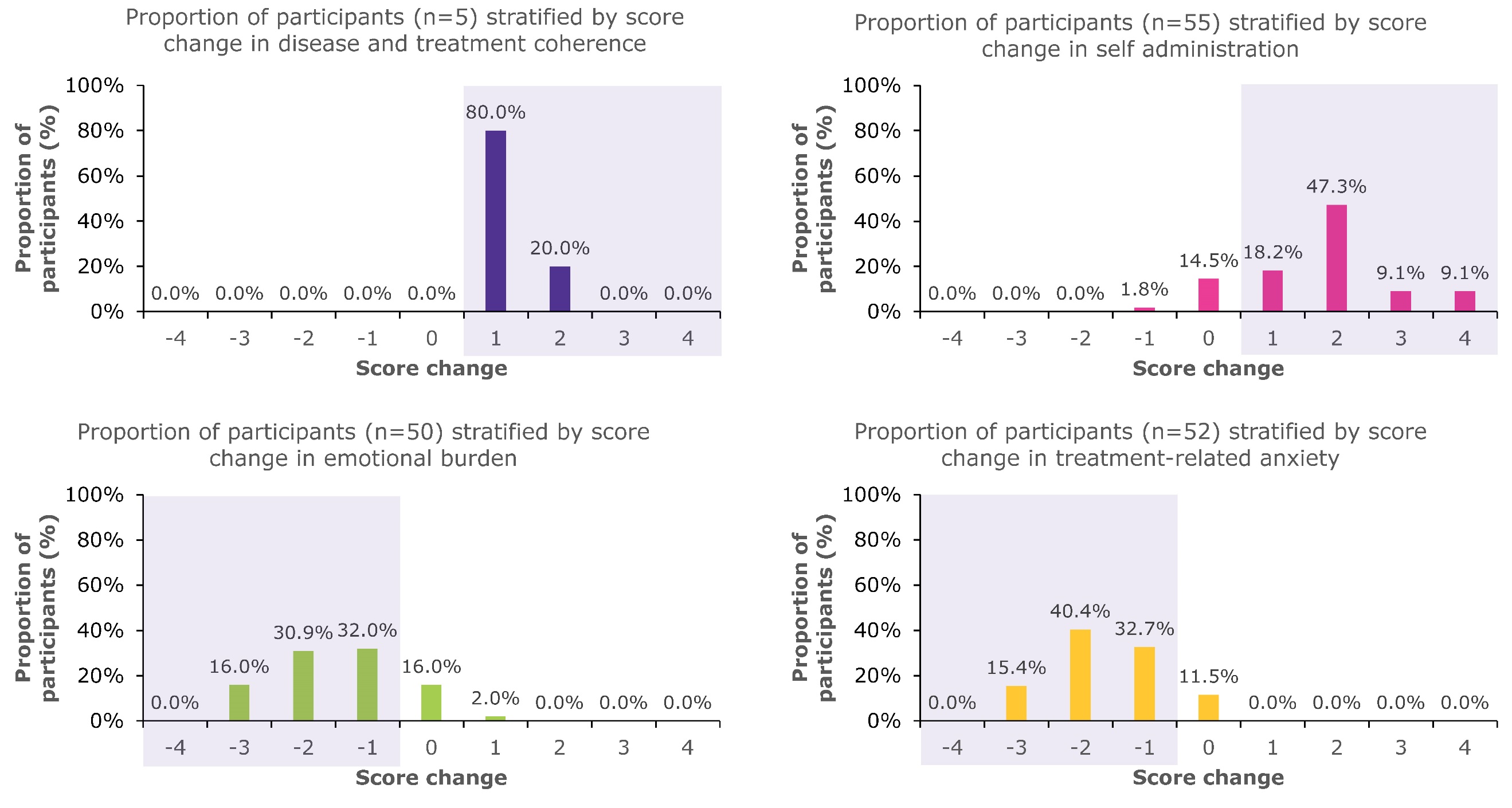
**

The shaded areas indicate beneficial changes in score

Supplementary Figure S3. Proportion of patients who were high-risk at baseline who had score changes in Taiwan

**
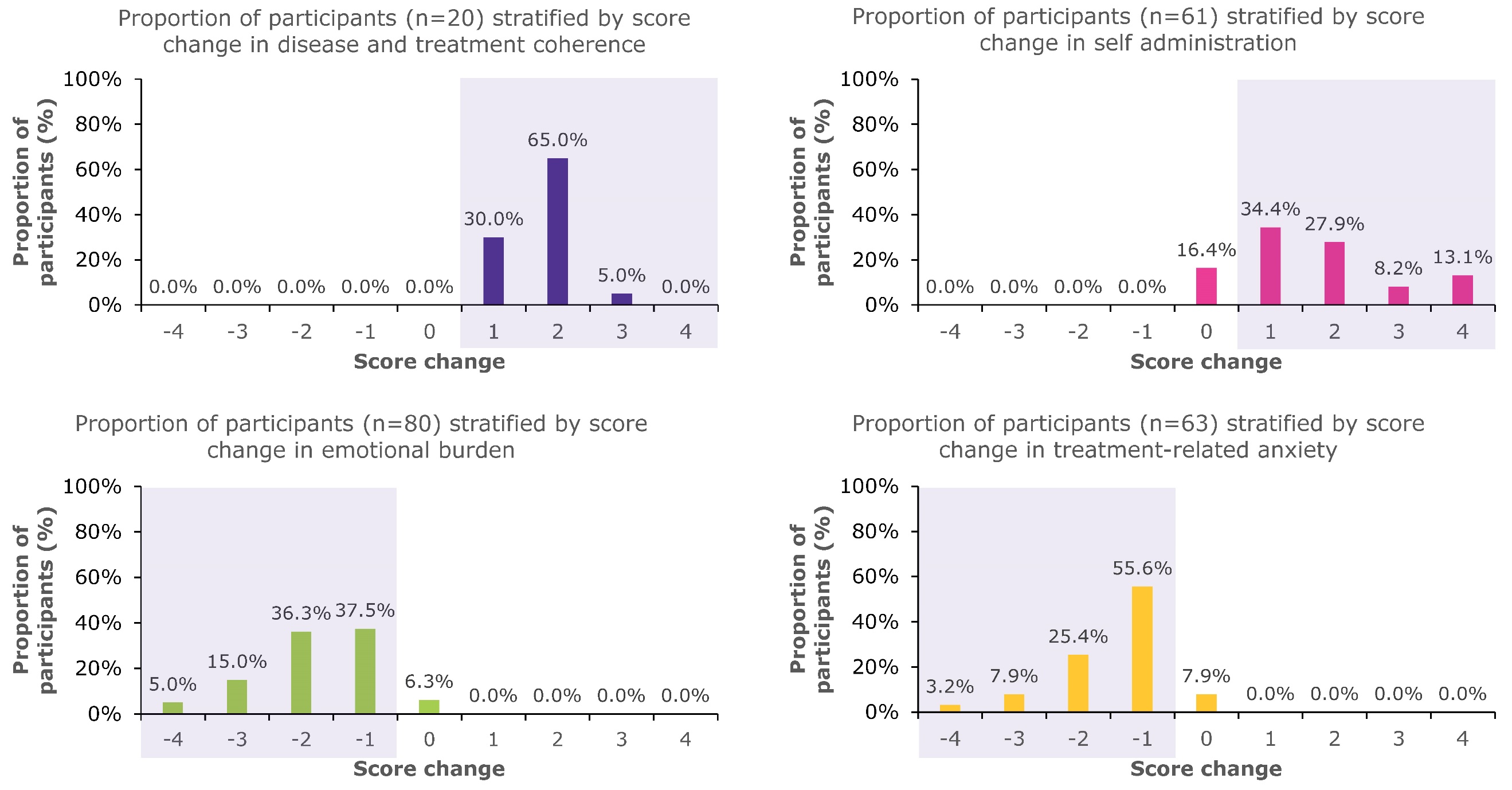
**

The shaded areas indicate beneficial changes in score

Supplementary Figure S4. Risk status of patients in the high-risk group for additional factors at baseline following TUITEK^®^ PSP in Taiwan


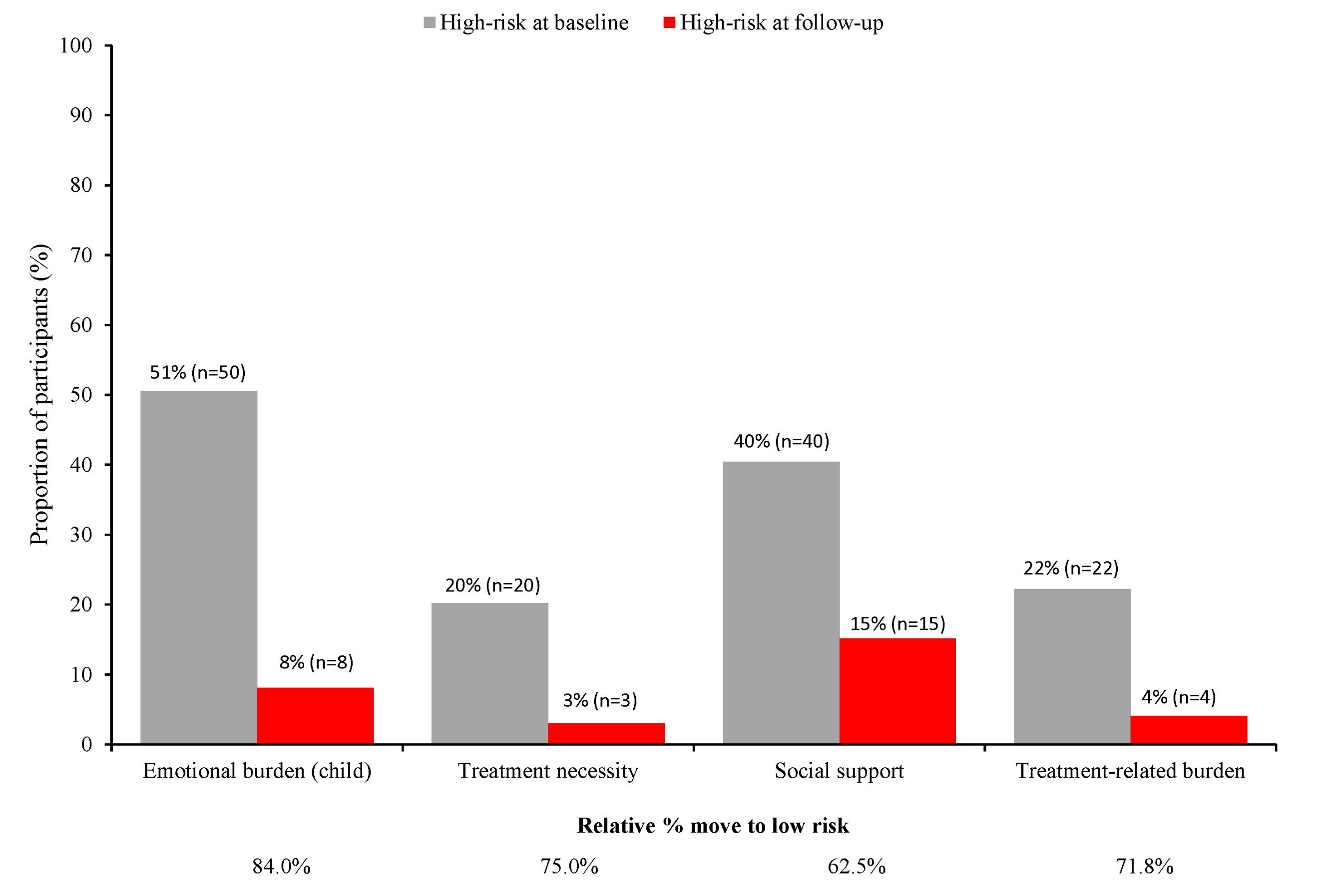


Data are only shown for patients at high risk at baseline, the remaining proportion of participants are those who are low risk at baseline.

Supplementary Figure S5. Proportion of patients who were high-risk at baseline for additional factors who had score changes in Taiwan

**
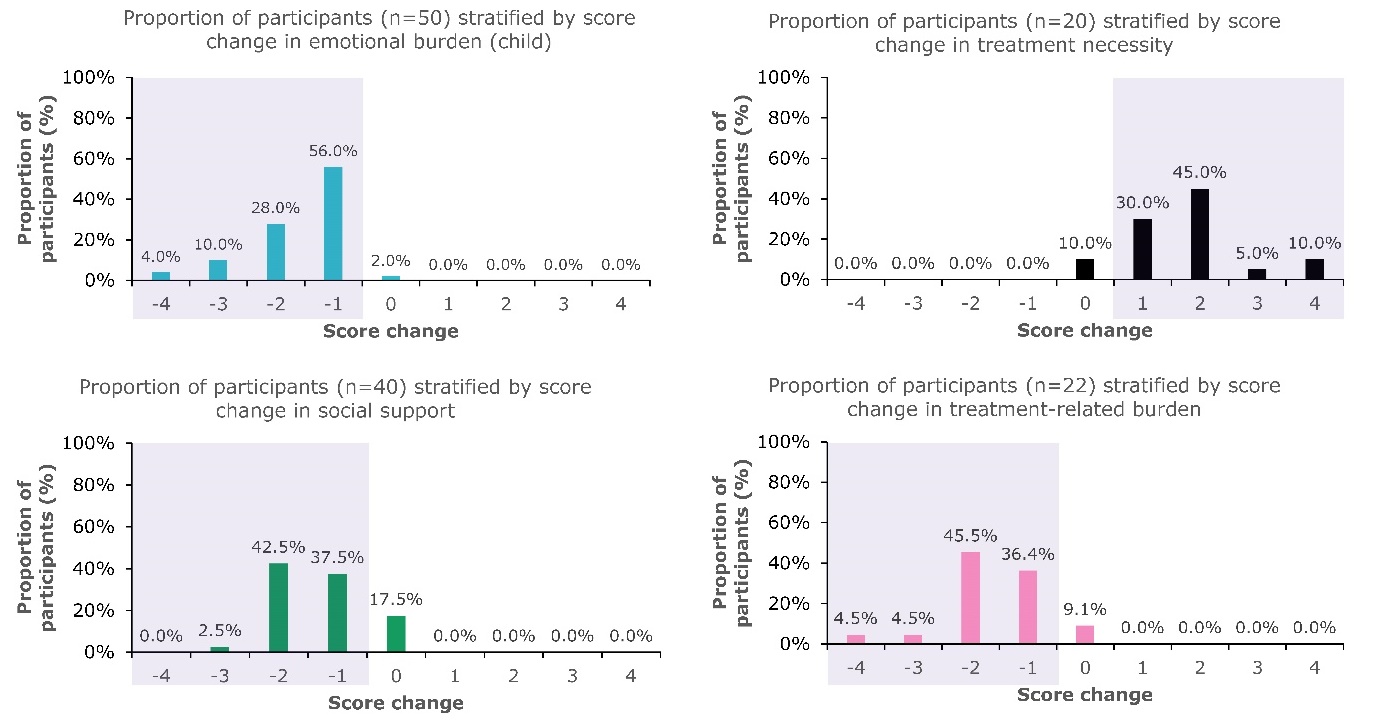
**

**Supplementary Table S1.** Personalization questionnaire delivered in Argentina.

| **Factor** | **Question** | **Scoring** | **Cut-off for**  **‘high-risk’** |
| --- | --- | --- | --- |
| **Disease and Treatment Coherence** | Do you understand your child's illness and treatment? | 5 - Understand very clearly  1 - Don't understand at all | **If** score of 1-3  **Then** caregiver receives call |
| **Disease Coherence** | How well do you understand your child's situation? | 5 - Understand very clearly  1 - Don't understand at all | **If** score of 1-3  **Then** caregiver receives call |
| **Treatment Coherence** | How well do you understand your child's treatment, and how to manage it? | 5 - Understand very clearly  1 - Don't understand at all | **If** score of 1-3  **Then** caregiver receives call |
| **Emotional Burden** | How does your child's situation affect you emotionally? | 5 - Extremely  1 - Not at all | **If** score of 3-5  **Then** caregiver receives call |
| **Self-Administration** | How comfortable do you feel giving your child responsibility for managing his or her illness and treatment? | 5 - Extremely comfortable 1 - not at all comfortable | **If** score of 1-3  **Then** caregiver receives call |
| **Treatment-related Anxiety** | How does your child's treatment affect you emotionally? | 5 - Extremely  1 - Not at all | **If** score of 3-5  **Then** caregiver receives call |

**Supplementary Table S2.** Personalization questionnaire delivered in South Korea.

| **Factor** | **Question** | **Scoring** | **Cut-off for**  **‘high-risk’** |
| --- | --- | --- | --- |
| **Disease Coherence** | How well do you think you understand (know) your child's overall condition (overall condition for growth hormone treatment)? | 5 - Understand very clearly  1 - Don't understand at all | **If** score of 1-3  **Then** caregiver receives call |
| **Treatment Coherence** | How well do you think you understand your child's medication (growth hormone) and how to administer it? |  | **If** score of 1-3  **Then** caregiver receives call |
| **Emotional Burden** | How much does your child's overall condition affect how you feel? (Example: Do you feel worried, angry, scared, upset, difficult, or burdened?) | 5 – Extremely affected emotionally  1 - Not affected emotionally at all | **If** score of 3-5  **Then** caregiver receives call |
| **Self-Administration** | How comfortable do you feel about your child taking responsibility for the management and injections of growth hormone treatment? | 5 - Extremely comfortable 1 - Not at all comfortable | **If** score of 1-3  **Then** caregiver receives call |
| **Treatment-related Anxiety** | How much does having to give your child a shot (or having your child get a shot) affect how you feel (e.g., worried, angry, scared, upset, or distressed)? , Do you feel burdened?) | 5 – Extremely affected emotionally  1 - Not affected emotionally at all | **If** score of 3-5  **Then** caregiver receives call |

**Supplementary Table S3**. Personalization questionnaire delivered in Taiwan.

| **Factor** | **Question** | **Scoring** | **Cut-off for**  **‘high-risk’** |
| --- | --- | --- | --- |
| **Disease and Treatment Coherence** | Do you understand your child’s treatment and disease status? | 5 - Understand very clearly  1 - Don't understand at all | **If** score of 1-3  **Then** caregiver receives call |
| **Emotional Burden** | How much does your child's disease (insufficient growth hormone secretion) affect your mood? (e.g. does it make you feel worried, angry, scared, annoyed or guilty) | 5 – Extremely emotionally affected  1 – Not at all affected emotionally | **If** score of 3-5  **Then** caregiver receives call |
| **Self-Administration** | Are you comfortable letting your child take charge of managing his or her own disease and administering growth hormone? | 5 - Extremely comfortable 1 - not at all comfortable | **If** score of 1-3  **Then** caregiver receives call |
| **Treatment-related Anxiety** | How concerned are you about your child's growth hormone treatment? (For example: side effects or drug injections | 5 - Extremely  1 - Not at all | **If** score of 3-5  **Then** caregiver receives call |
| **New questions** |  |  |  |
| **Emotional Burden (child)†** | How much is your child's mood affected by his/her medical condition? | 5 - Extremely  1 – Not at all | **If** score of 3-5  **Then** caregiver receives call |
| **Treatment Necessity†** | Can your child use growth hormone treatment to improve health and maintain good health? | 5 - Completely  1 - Not at all | **If** score of 1-3  **Then** caregiver receives call |
| **Social Support†** | Is it easy to find appropriate social support for your child's condition and treatment? | 5 – Not at all easily 1 – Extremely easily | **If** score of 1-3  **Then** caregiver receives call |
| **Treatment-related burden†** | How much of an impact does having your child's medication injections have on your daily life? | 5 - Extremely  1 - Not at all | **If** score of 3-5  **Then** caregiver receives call |
